# Pharmacological Inhibition of CDK8 in Triple-Negative Breast Cancer Cell Line MDA-MB-468 Increases E2F1 Protein, Induces Phosphorylation of STAT3 and Apoptosis

**DOI:** 10.3390/molecules25235728

**Published:** 2020-12-04

**Authors:** Jensen M. Spear, Zhixin Lu, Wade A. Russu

**Affiliations:** Department of Pharmaceutics and Medicinal Chemistry, Thomas J. Long School of Pharmacy, University of the Pacific, Stockton, CA 95211, USA; j_spear2@u.pacific.edu (J.M.S.); z_lu3@u.pacific.edu (Z.L.)

**Keywords:** CDK8, E2F1, STAT3, MDA-MB-468 cells, breast cancer, quinazoline, piperazinylpyrimidine

## Abstract

Cyclin-dependent kinase 8 (CDK8) has been identified as a colon cancer oncogene. Since this initial observation, CDK8 has been implicated as a potential driver of other cancers including acute myelogenous leukemia (AML) and some breast cancers. Here, we observed different biological responses to CDK8 inhibition among colon cancer cell lines and the triple-negative breast cancer (TNBC) cell line MDA-MB-468. When treated with CDK8 inhibitor **4**, all treated cell lines responded with decreased cell viability and increased apoptosis. In the MDA-MB-468 cell line, the decrease in cell viability was dependent on increased phosphorylation of signal transducer and activator of transcription 3 (STAT3), which is not observed in the colon cancer cell lines. Furthermore, increased STAT3 phosphorylation in **4** treated MDA-MB-468 cells was dependent on increased transcription factor E2F1 protein. These results are consistent with previous reports of exogenous expression of E2F1-induced apoptosis in MDA-MB-468 cells.

## 1. Introduction

Cyclin-dependent kinase 8 (CDK8) is a positive regulator of the cell cycle and has been described as an oncogene in the context of chromosomal amplification in colon cancer cell lines [1,2]. Amplification of 13q12 occurs in a significant fraction of colon cancer and CDK8 was identified by an RNA interference screen as a relevant gene target and likely oncogene. Knockdown of CDK8 in colon cancer cell lines led to decreased proliferation. Furthermore, transfection of NIH3T3 cells with CDK8 resulted in cellular transformation, while transfection with a kinase dead mutant did not. CDK8 was also shown to regulate the β–catenin transcriptional activity. Due to the role of many CDK family members in the regulation of the cell cycle or regulation of transcription, these proteins have become of interest as potential drug targets of cancer therapeutics [3,4].

CDK8 regulates gene transcription as a part of the mediator complex. CDK8 has been demonstrated to associate with mediator of RNA polymerase II transcription subunits 12 and 13 (MED12 and MED13), and cyclin-C (CCNC) as a mediator complex subunit that can phosphorylate RNA polymerase II (RNAP II) [5,6]. Besides being a part of the mediator complex, CDK8 can regulate gene transcription through modulating chromatin structure by phosphorylating histone H3 [7]. CDK8 can also directly phosphorylate transcription factors E2F1 and STAT1 [8,9]. One hypothesis is that CDK8 phosphorylation of E2F1 attenuates its repressive effect on β–catenin expression, which contributes to colon carcinogenesis. CDK8 has also become a target of interest in other cancers such as acute myologenous leukemia (AML), melanoma, and prostate [10,11,12,13].

**Figure 1:**
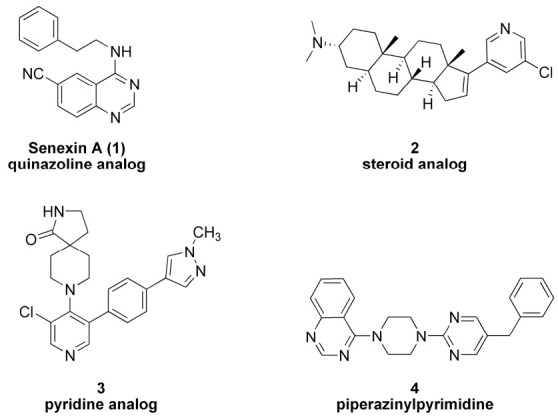
CDK8 inhibitor structures of diverse chemotypes.

Since the discovery of the oncogenic property of CDK8, there has been considerable effort to develop potential small molecule drugs that can inhibit its kinase activity. Chemotypes discovered to inhibit CDK8 kinase activity range from simple quinazoline senexin A (**1**), steroid analogs (**2**), pyridine analogs (**3**) to piperazinylpyrimidines (**4**) [14,15,16,17]. These small molecules bind to the adenosine triphosphate (ATP)-binding site of CDK8. Figure 1 demonstrates the diversity of chemotypes that can inhibit CDK8. We selected inhibitor **4** for our studies because of its potent cytotoxicity against MBA-MB-468 cell line [17].

Here, we describe the preliminary results of our investigations aimed at understanding the cell biological effects of CDK8 inhibitor **4** on triple-negative breast cancer (TNBC) cell line MDA-MB-468 compared to both APC mutant and β–catenin mutant colon cancer cell lines Colo205 and HCT116.

## 2. Results

### 2.1. Effects of ***4*** on Cell Viability

Since the initial suggestion that CDK8 is an oncogene relevant to colon cancer and may play a role in other types of tumors including breast tumors, we attempted to compare the effects of CDK8 inhibitor **4** on colon cancer cell lines and the TNBC cell line MDA-MB-468.

**Figure 2:**
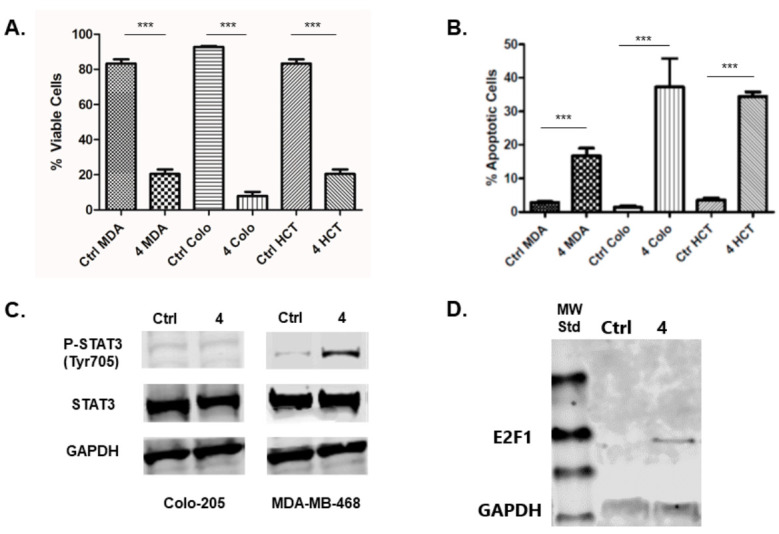
(**A**) Effect of treatment with 10 µM inhibitor **4** (72 h) on cell viability of MDA-MB-468 (MDA), Colo205 (Colo) and HCT116 (HCT) cells compared to vehicle-treated control (Ctrl) cells. (**B**) Effect of treatment with 10 µM inhibitor **4** (48 h) on apoptosis of MDA-MB-468 (MDA) and Colo205 (Colo) cells compared to vehicle-treated control (Ctrl) cells. (**C**) Effect of treatment with 10 µM inhibitor **4** (24 h) on STAT3 phosphorylation status in MDA-MB-468 (MDA) and Colo205 (Colo) cells compared to vehicle treatment. (**D**) Effect of treatment with 10 µM inhibitor **4** (24 h) on E2F1 protein expression in MDA-MB-468 cells compared to vehicle-treated control (Ctrl). *** *p* < 0.001 (very significant).

The treatment of colon cancer cell lines HCT116 and Colo205 with inhibitor **4** results in a decrease in cell viability and induction of apoptosis in these cell lines (Figure 2A,B). This result is consistent with numerous literature reports that investigate cell biological effects of CDK8 inhibitors used as chemical probes or drug leads [16,18,19,20,21]. The same treatment regimen resulted in a decrease in cell viability and the induction of apoptosis in TNBC cell line MDA-MB-468 (Figure 2A,B).

### 2.2. Effects of Inhibitor ***4*** on β–Catenin, STAT1, STAT3 and E2F1 Proteins

It has been demonstrated that colon cancer cell lines treated with CDK8 RNAi result in decreased cellular levels of β–catenin [2]. Likewise, treatment of colon cancer cell line Colo205 with CDK8 inhibitor **4** results in a dramatic depletion of β–catenin protein. The amount of β–catenin protein observed when TNBC cell line MDA-MB-468 is treated with inhibitor **4** did not appear to change significantly (Figure S1).

The phosphorylation status of STAT1 protein is a robust pharmacodynamic marker for CDK8 inhibition [9]. Treatment of the colon cancer cell line Colo 205 and the TNBC cell line with inhibitor **4** resulted in decreased STAT1 phosphorylation (pSTAT1), indicating inhibition of CDK8 in all these cell lines (Figure S1), as expected. In contrast, STAT3 phosphorylation (pSTAT3) status was unchanged in the Colo205 cancer cell line, while being elevated in the TNBC cell line upon treatment with CDK8 inhibitor **4** (Figure 2C).

We next looked at the effects of inhibitor **4** on E2F1 protein, specifically in the MDA-MB-468 cell line. The treatment of MDA-MB-468 cell with inhibitor **4** resulted in increased E2F1 protein in this cell line. In non-treated control cells, E2F1 is difficult to detect via Western blot, while in the cells treated with inhibitor **4,** the protein is obviously present (Figure 2D).

### 2.3. Effects of E2F1 RNAi on STAT3 Protein

**Figure 3:**
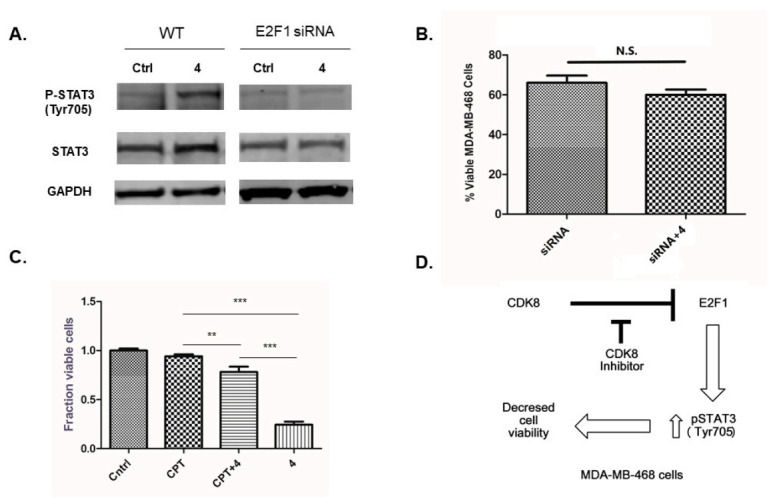
(**A**) Comparison of treatment with 10 µM inhibitor **4** (24 h) on STAT3 phosphorylation in wild-type (WT) MDA-MB-468 cells and MDA-MB-468 E2F1 knockdown cells (E2F1 siRNA). (**B**) Effect on MDA-MB-468 cell viability of E2F1 knockdown alone (siRNA) and with 10 µM inhibitor **4** (72 h) (siRNA + **4**). (**C**) Effect on MDA-MB-468 cell viability of treatment (10 µM, 72 h) with STAT3 phosphorylation inhibitor cryptotanshinone (CPT), CPT + inhibitor **4** co-treatment, and treatment with **4** alone compared to vehicle treated control (Cntrl). (**D**) Proposed CDK8 inhibitor mechanism. N.S. *p* > 0.05 (not significant), ** *p* < 0.005 (significant), *** *p* < 0.001 (very significant).

To assess whether phosphorylation of STAT3 was dependent on E2F1, we compared STAT3 phosphorylation in MDA-MB-468 cells treated with siRNA targeting E2F1 in the presence and absence of inhibitor **4**. In these experiments, targeting E2F1 with siRNA prevented the increase in phosphorylation of STAT3 due to treatment with inhibitor **4** (Figure 3A). Additionally, there was not a significant difference between the viability of MDA-MB 468 cells treated with E2F1 siRNA and cells treated with both E2F1 siRNA and inhibitor **4**, suggesting that the upregulation of the E2F1 protein is necessary for the cytotoxic effects of inhibitor **4** (Figure 3B).

In order to assess whether STAT3 activation was linked to a decrease in the cell viability of MDA-MB-468 treated with inhibitor **4**, MDA-MB-468 cells were co-treated with inhibitor **4** and a STAT3 Tyr705 phosphorylation inhibitor cryptotanshinone (CPT), and compared to cells treated with inhibitor **4** alone, CPT alone and non-treated cells. The STAT3 Tyr705 phosphorylation inhibitor attenuated the effects of inhibitor **4** (Figure 3C), suggesting that phosphorylation of STAT3 at Tyr705 is necessary for the cytotoxic effects of inhibitor **4**.

## 3. Discussion

The identification of CDK8 as a colon cancer oncogene and regulator of β–catenin protein has spurred intense interest in the potential involvement of CDK8 in other types of cancer including breast cancer. It is interesting to note that CDK8 is amplified in a significant number of breast cancers, but low β–catenin levels are correlated with poor prognosis [22]. This suggests that the roles of CDK8 and β–catenin differ between colon cancers and breast cancers. Here, we have demonstrated that pharmacological inhibition of CDK8 in colon cancer cell lines HCT116 and Colo205 resulted in depletion of β–catenin protein while the same treatment of breast cancer cell line MDA-MB-468 did not appear to change β–catenin content (Supplemental Figure S1). In the colon cancer cell lines, these results are consistent with the reported depletion of β–catenin due to CDK8 knockdown [2]. The contrast between the colon cancer cell lines and the breast cancer cell line results suggest that CDK8 may not be a major regulator of β–catenin in MDA-MB-468 cells, or a compensatory mechanism exists.

A consistent pharmacodynamic marker of CDK8 inhibition is the phosphorylation status of STAT1. CDK8 inhibition results in the decrease or suppression of pSTAT1 [9]. Our results are consistent with previous reports, whereas we have shown that treatment with inhibitor **4** resulted in a decrease in pSTAT1 in both the colon cancer cell lines as well as the MDA-MB-468 cell line (Supplemental Figure S1). In contrast to this result, the phosphorylation state of STAT3 was either unchanged, as in the colon cancer cell lines, or increased, as in the case of MDA-MB-468 cell line. This is a very interesting cellular context dependent pharmacologic result. Furthermore, we have shown that inhibition of STAT3 phosphorylation attenuated the decrease in cell viability of MDA-MB-468 cells when treated with inhibitor **4**. This is a particularly interesting result considering the STAT family’s association with the expression of various proteins related to the process of apoptosis [23].

CDK8 is known to phosphorylate E2F1 and to repress its transcriptional activity and this results in upregulated β–catenin protein in colon cancer cells [24]. Knockdown of E2F1 in osteosarcoma cells results in reduced phosphorylation of STAT3 [25]. This establishes a possible link between E2F1 activity and phosphorylation of STAT3. Knockdown of E2F1 in prostate cancer cell line DU145 results in the reduced expression of cytokines including Il-6. When treated with IL-6 family member oncostatin M (OSM), MDA-MB-468 cells respond with increased levels of phosphorylated STAT3 and undergo apoptosis [26]. Furthermore, overexpression of E2F1 in MDA-MB-468 breast cancer cells induces apoptosis [27]. We have demonstrated that treatment of MDA-MB-468 cells with CDK8 inhibitor **4** results in increased E2F1 protein, increased STAT3 phosphorylation, decreased cell viability and induction of apoptosis.

In the context of breast cancer, CDK8 along with S-phase kinase-associated protein 2 (Skp2) has been shown in tissue samples to positively correlate with stage of breast cancer [28]. In the context of estrogen receptor positive (ER+) breast cancer, it has been shown that CDK8 inhibition suppressed estrogen induced gene transcription through reduced RNAPII phosphorylation, suggesting a potential role of CDK8 inhibitors with antiestrogen therapy [29]. Furthermore, miRNA-mediated knockdown of CDK8 reduces the proliferation and migration of breast cancer cells [30]. In chem-naïve breast cancer patients (sub-class unspecified), tumor specimens demonstrated lower CDK8-targeting micro-RNA than in adjacent tissue [31]. The relevance of CDK8 in TNBC specifically has not been addressed directly. However, when considering CDK8 ability to suppress E2F1 activity, there are some intriguing reports to consider.

It has been shown that TNBC cell lines MDA-MB-468 and MDA-MB-436 express low levels of E2F1 and that when E2F1 is overexpressed in these cell lines, cell viability is reduced and apoptosis is induced [27]. This study used exogenous expression of E2F1 and not reactivation of endogenous E2F1. Another report determined that TNBC patients that showed the induction of apoptosis when treated with doxorubicin also showed an upregulation in E2F1 in biopsied tumors and that non-responders to treatment did not show upregulated E2F1 [32]. This report demonstrates that upregulation of E2F1 as result of chemotherapy in TNBC patients is associated with chemotherapy positive response. Here, we have observed in TNBC MDA-MB-468 cells that treatment with a CDK8 inhibitor upregulates E2F1 and is associated with increased STAT3 phosphorylation (pSTAT3 Tyr705), decreased cell viability and induction of apoptosis.

We have shown that pharmacologic inhibition of CDK8 with inhibitor **4** causes depletion of β–catenin protein in Colo205, HCT116 but not in the MDA-MB-468 cell line. This treatment also causes a decrease in cell viability with the induction of apoptosis in all three cell lines. Triple-negative breast cancer cells, MDA-MB-468 cells, upregulate E2F1 protein when treated with inhibitor **4**. It is interesting to note that knockdown of CDK8 in colon cancer cell lines HCT116, LOVO, and SW480 does not appear to affect E2F1 protein levels, but does affect E2F1 transcriptional activity [33].We have also demonstrated that the decrease in cell viability of MDA-MB-468 cells treated with inhibitor **4** is dependent on the induction of STAT3 phosphorylation, and this effect is not observed in the two colon cancer cell lines used in this study. Figure 3D represents a proposed mechanism by which CDK8 inhibition decreases cell viability of MDA-MB-468.

An understanding of the cell-context-dependent link between CDK8 inhibition, increased E2F1 protein and induction of STAT3 phosphorylation and cell viability could provide an opportunity for the establishment of a biomarker for the identification of tumors likely to respond to CDK8-targeted therapy. Additionally, such new knowledge may provide opportunities to identify new drug targets. We are continuing this mechanistic investigation and will report our results in due course.

## 4. Materials and Methods

### 4.1. Cell Lines, Chemicals and Antibodies

MDA-MB-468 human breast cells, COLO-205 human colon cells and HCT-116 human colon cells were obtained from ATCC, USA. MDA-MB-468 and COLO-205 were grown in Gibco^®^ RPMI-1640 media (Thermo Fisher Scientific, Walthan, MA, USA), HCT-116 was grown in McCoy’s 5a media (Hyclone, Logan, UT, USA). All media were supplemented with 10% FBS (Gemini Biological Products, Calabasas, CA, USA), 1% Antibiotic–Antimycotic mixture of Penicillin-G, Streptomycin sulfate and Amphotericin B (Thermo Fisher Scientific, Walthan, MA, USA). The cells were maintained in a humidified incubator at 37 °C with 5% CO_2_. Antibodies to E2F1, STAT1, *p*-STAT1 (Ser727), STAT3, *p*-STAT3 (Tyr705), β-catenin, and GAPDH were obtained from Cell Signaling Technology, Danvers, MA. E2F1 siRNA and siRNA transfection reagent were purchased from Santa Cruz Biotechnology (Dallas TX, USA) and used according to the manufacturer’s protocol. Cryptotanshinone (CPT) was purchased from Selleck Chemicals (Houston, TX, USA) and **4** was prepared and purified to >97% according to the literature procedure [17].

### 4.2. Cell Viability

Cells were treated with 0.1% DMSO (vehicle) or 10 µM **4** or 10 µM cryptotanshinone or combination of both **4** and cryptotanshinone for 72 h. Final DMSO concentration was 0.1% in all cases. Floating cells and adherent cells were collected after the various treatments. Cells were washed twice with 1 × PBS and re-suspended in fresh media for cell viability assay. In the case where E2F1 siRNA was used as treatment, transfection was performed according to the manufacturer’s protocol. Cell viability was measured with the Muse Cell Analyzer (Millipore, Hayward, CA, USA) using the Muse Count and Viability Kit (Millipore, Hayward, CA, USA) according to manufacturer’s protocol. Data were collected from three independent experiments.

### 4.3. Apoptosis Assay

Cells were treated with 0.1% DMSO (vehicle) or 10 µM **4** for 24 h. Detached and adherent cells were collected and treated according to the manufacturer’s protocol using Luminex Muse Annexin V and Dead Cell Kit (Luminex Corp, Austin, TX, USA). The events for live, dead, early and late apoptotic cells were counted with the Muse Cell Analyzer (Millipore, Hayward, CA, USA). Data were collected from three independent experiments.

### 4.4. Immunoblotting

Cells were collected and washed twice with ice-cold 1 × HBSS, then lysed in Cell Lysis Buffer (Cell Signaling Technology, Danvers, MA, USA) supplemented with protease inhibitor (Sigma, Ronkonkoma, NY, USA) on ice for 10 min before scrapping. Cell lysates were then centrifuged for 10 min at 13,000 rpm at 4 °C. The protein concentration of lysates was determined by BCA protein assay kit (Thermo Fisher Scientific, Walthan, MA, USA) and the lysates were adjusted with a lysis buffer. Proteins were separated using SDS-PAGE and subsequently transferred to nitrocellulose membranes. The blots were blocked with 5% BSA (20mM Tris HCL, pH 7.5, 137 mM NaCl and 0.05% Tween-20) at room temperature for 1 h. Incubation with specific primary antibodies was performed in blocking buffer overnight at 4 °C. After washing with TBST, the blots were incubated with secondary antibody (IRDye 800CW Donkey anti-rabbit 926–32,213, LI-COR Biosciences, Lincoln, NE, USA) for 1 h. To ensure equal protein loading, GAPDH was used as an internal control. The protein bands were detected and quantified with the Odyssey infrared imaging system (LI-COR Biosciences, Lincoln, NE, USA). Data were collected from three independent experiments.

### 4.5. Statistics

Statistical analysis was performed using GraphPad Prism (GraphPad, La Jolla, CA, USA) employing either *t*-test or ANOVA. *p* < 0.05 is considered significant.

## Supplementary Materials

The following is available online. Figure S1: Representative Western blots for β-catenin and phosphorylated STAT1 (*p*-STAT1 (Ser 727)) from cell lines MDA-MB-468, COLO-205, HCT-116 that were vehicle-treated (control) or treated with **4** (10 µM) for 24 h.
