# Supplementary material for: Pharmacological Inhibition of CDK8 in Triple-Negative Breast Cancer Cell Line MDA-MB-468 Increases E2F1 Protein, Induces Phosphorylation of STAT3 and Apoptosis
Source: Molecules. 2020 Dec 4;25(23):5728. doi: 10.3390/molecules25235728 (PMC7730658; doi:10.3390/molecules25235728)
Supplement: Supplementary file 1 [file molecules-25-05728-s001.pdf]

Pharmacological inhibition of CDK8 in triple negative breast cancer cell line MDA-MB-468 increases E2F1 protein, induces phosphorylation of STAT3 and apoptosis.

Jensen M. Spear, Zhixin Lu, and Wade A. Russu

Department of Pharmaceutics and Medicinal Chemistry, Thomas J. Long School of Pharmacy, University of the Pacific, Stockton, CA 95211

Supplementary Information

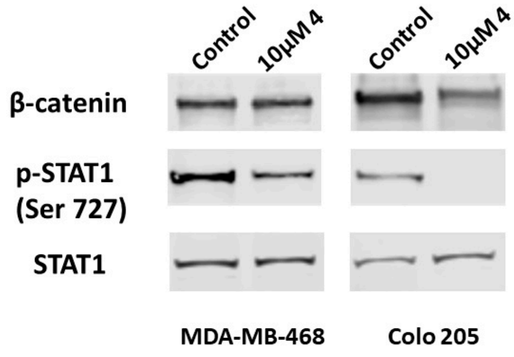

Figure S1. Representative western blots for β-catenin and phosphorylated STAT1 (p-STAT1 (Ser 727)) and total STAT1 from the indicated cell lines (MDA-MB-468, Colo-205) that were vehicle treated (control) or treated with **4** (10 µM) for 24h.
